# Life-Threatening Pneumomediastinum Following Tracheal Perforation as a Complication of Total Thyroidectomy: A Case Report

**DOI:** 10.7759/cureus.104586

**Published:** 2026-03-02

**Authors:** Chiraz C Halwani, Mohamed Anas Ammar, Rihem Ksouri, Sana Boughariou, Sonia Esseghaier

**Affiliations:** 1 Department of Otorhinolaryngology, Military Hospital of Tunis, Tunis, TUN; 2 Department of Otolaryngology - Head and Neck Surgery, Military Hospital of Tunis, Tunis, TUN; 3 Department of Anaesthesiology, Military Hospital of Tunis, Tunis, TUN; 4 Department of Reanimation, Military Hospital of Tunis, Tunis, TUN; 5 Department of Radiology, Military Hospital of Tunis, Tunis, TUN

**Keywords:** otolaryngology, pulmonary critical care, rare surgical complication, thyroidectomy complications, tracheal perforation

## Abstract

Total thyroidectomy, a common surgical procedure, is generally safe with a low morbidity and mortality rate. While complications such as hematoma and recurrent laryngeal nerve injury are well recognized, tracheal perforation is a rare but life-threatening event. We discuss the risk factors associated with tracheal perforation during thyroidectomy and review current best practices for its management. This case report describes a 55-year-old woman who experienced a rare but critical complication following total thyroidectomy: tracheal perforation leading to bilateral pneumothorax and mediastinal emphysema. The successful management of this patient, involving prompt reoperation, tracheostomy, chest tube placement, and antibiotic treatment for subsequent cervicomediastinal cellulitis, highlights the importance of early recognition and intervention in managing this life-threatening event.

## Introduction

Total thyroidectomy is a frequently performed surgical procedure for the treatment of thyroid cancer, multinodular goiter, and other thyroid pathologies [1]. Although highly successful, the procedure is not without risks, which can include common complications such as hematoma and recurrent laryngeal nerve injury, as well as rarer complications such as tracheal perforation [2]. This complication is serious due to the risk of life-threatening airway compromise and requires immediate diagnosis and prompt treatment. Through this report, we discuss the risk factors that can lead to such a rare complication and review management strategies based on recent literature.

## Case presentation

A 55-year-old woman presented with a large goiter that had recently caused dysphagia, but she reported no difficulty breathing. Her medical history included hypertension and severe obesity (body mass index: 35 kg/m²). Upon examination, a low, firm, painless anterior cervical swelling was noted, although the lower margin could not be palpated. A pharyngolaryngeal examination revealed normal, mobile vocal cords. Thyroid function tests were within normal limits.

Cervical ultrasound identified a multinodular substernal goiter, featuring a dominant 45 mm solid nodule in the left lobe, classified as EUTIRADS 3, with the lower margin not visualized. A CT scan indicated a deviation of the tracheoesophageal tract but no vascular adhesions.

Total thyroidectomy was performed through a transcervical incision. During the operation, the nodule was difficult to expose due to its adhesion to the lateral tracheal wall. The procedure was complicated by a 0.8 cm tracheal perforation at the fourth tracheal ring, which was repaired using a sternocleidomastoid muscle flap. After extubation, the patient’s oxygen saturation was 100%, and she was fully conscious and pain-free.

However, two hours postoperatively, she suddenly developed dyspnea and subcutaneous emphysema following a cough. Her oxygen saturation rapidly dropped to 80%, necessitating 10-15 L/minute of oxygen via a face mask to maintain her saturation above 90%. She was immediately taken back to the operating room.

**Figure 1:**
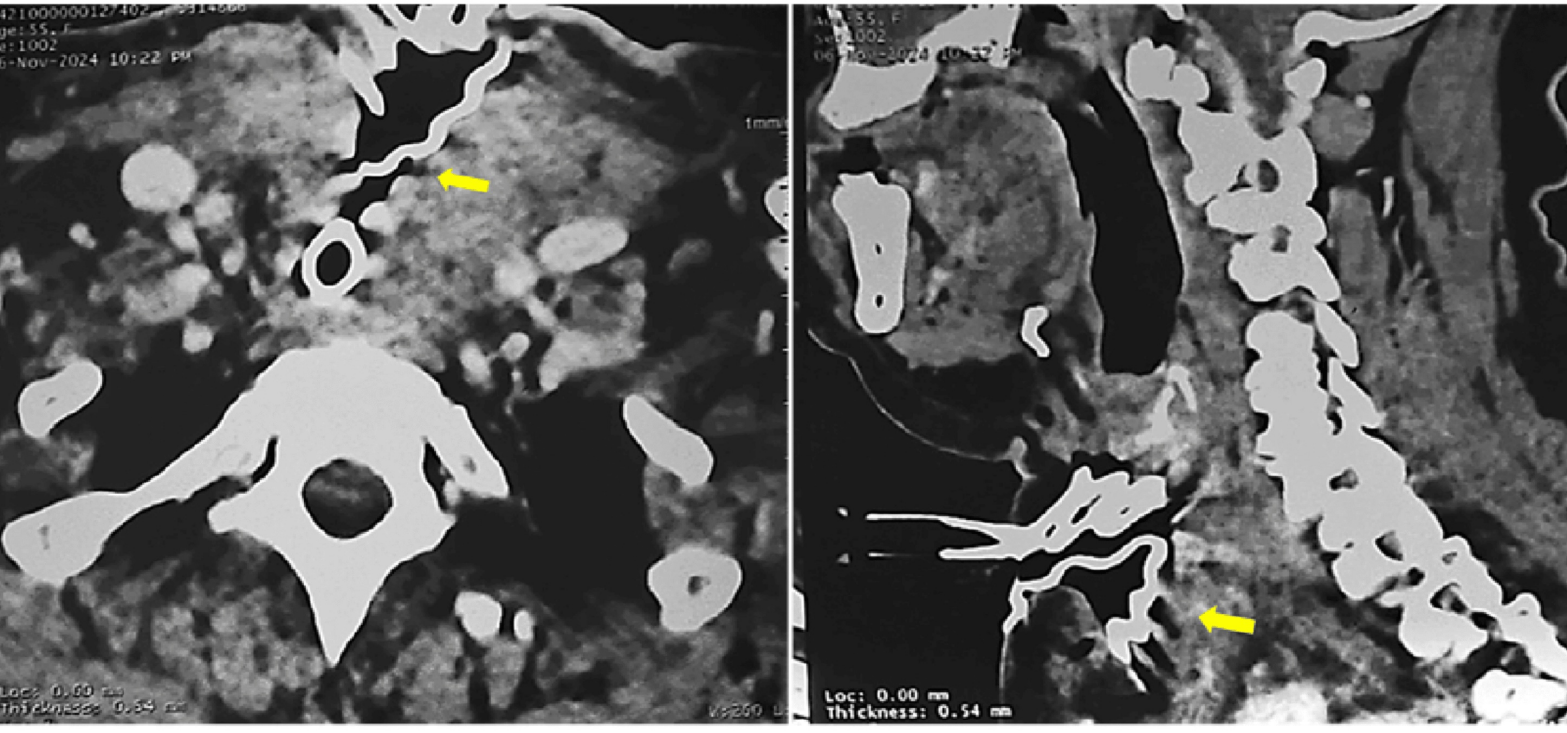
Tracheostomy orifice facing a large tracheal wound, drainage blade in place (yellow arrow), with significant cervico-mediastinal emphysema.

The ENT team reopened the surgical site and found no hematoma. As her oxygen saturation plummeted to 40% and breath sounds were absent bilaterally, bilateral pneumothorax was suspected. Emergency anesthesia was induced, but tracheal intubation proved very difficult (Cormack-Lehane grade IV). The team extended the tracheal injury and performed a tracheostomy, successfully placing a size 7 Tracheoflex tube. A follow-up cervico-mediastinal CT scan confirmed bilateral pneumothorax (Figure 1).

The patient was then transferred to the intensive care unit, where bilateral thoracostomy tubes (Pleurocath drains) were inserted. Follow-up chest radiographs showed resolution of the pneumothorax. After her respiratory status stabilized and radiological confirmation was obtained, sedation was stopped, and the patient was successfully decannulated. The thoracostomy drains were removed three days later, and she was transferred back to ENT care.

On postoperative day five, she developed pneumonia and cervicomediastinal cellulitis caused by *Klebsiella pneumoniae*, which was successfully treated with antibiotics. She was discharged home in good condition on day 21. Histopathology confirmed the presence of a benign goiter.

## Discussion

Total thyroidectomy is one of the safest surgical procedures performed today, with some of the lowest rates of complications, such as hematoma, recurrent laryngeal nerve injury, hypocalcemia, and wound infections [2]. However, rare but serious complications such as tracheal perforation can occur, with an estimated incidence of approximately 0.06% according to Gosnell et al. [3].

In a review published in 2023, Heo et al. [4] reported only about 25 cases in the literature, most of which were delayed perforations. These delayed forms represent a different etiology compared with the intraoperative perforation observed in our patient. Stevens et al. described such a case of delayed tracheal perforation, underscoring the need for rapid airway control when such an injury is suspected [5].

In our case, the decision to perform an emergency tracheostomy was justified, as it secured the airway and likely prevented progression to life-threatening complications such as mediastinitis or respiratory collapse [6]. Early recognition is critical to prevent morbidity and mortality. Several mechanisms may contribute to tracheal injury during thyroidectomy. These include dissection of the lateral and/or posterior tracheal wall near the recurrent laryngeal nerve entry point [7], devascularization of the posterior-lateral tracheal wall due to the use of diathermy [8], inadvertent suturing of vessels in that area, and aggressive dissection of the thyroid isthmus from the trachea [7]. Furthermore, anatomical variations and the presence of large substernal goiters can increase the risk of tracheal injury due to distortion and adherence of the gland to adjacent structures.

Detection of tracheal rupture can be challenging, especially when the defect is small or when symptoms are initially subtle. Intraoperative testing by irrigating the tracheal wound with saline before closure is recommended to detect potential air leaks early [8]. Postoperative signs such as subcutaneous emphysema, respiratory distress, or sudden hypoxia should prompt immediate investigation for possible tracheal injury or pneumothorax [5]. In the present case, a perforation measuring less than 1 cm was likely responsible for the bilateral pneumothorax. The coughing episode may have triggered a high-pressure air leak through the perforated trachea. Similar cases have reported pneumothorax resulting from difficult dissection near the pleura and mediastinum [9]. This highlights the importance of gentle tissue handling and awareness of the close anatomical relationships during surgery. Most patients recover uneventfully with primary repair of the trachea [3].

Typically, tracheal perforations are repaired by suturing the edges with absorbable sutures, often reinforced with adjacent strap muscle flaps and fibrin glue for additional support [3]. In cases of extensive injury or respiratory compromise, as in our patient, tracheostomy may be required to secure the airway and allow for healing. Preventive measures to reduce the risk of tracheal injury include thorough preoperative imaging, such as a contrast-enhanced cervico-mediastinal CT scan, to assess tracheal anatomy and adherence. Careful dissection with minimal use of energy devices near the trachea is also recommended. Additionally, maintaining a high level of suspicion in cases of difficult anatomy or prior surgery is crucial.

Multidisciplinary management involving ENT surgeons, anesthesiologists, and intensivists is essential for optimal outcomes in complex cases [5]. In our case, after emergency tracheostomy and pleural drainage, the patient’s respiratory status stabilized, and the tracheal wall was left to heal spontaneously following decannulation. The favorable outcome underscores the importance of prompt diagnosis and tailored management strategies.

## Conclusions

Tracheal injury during thyroidectomy, although exceedingly rare, poses significant clinical risks. Tracheal perforation, whether an intraoperative event or postoperative complication, is well described and requires prompt, appropriate, and multidisciplinary management to prevent prolonged hospitalization, serious morbidity, or even death. This case highlights the critical need for vigilant monitoring of airway complications following thyroidectomy, particularly in high-risk patients. Timely intervention plays a pivotal role in minimizing the morbidity and mortality associated with tracheal perforation.
